# Enhanced optoelectronic performance of MWCNT/n-Si Schottky photodetectors *via* cryogenic treatment of carbon nanotubes

**DOI:** 10.1039/d6ra04019e

**Published:** 2026-07-17

**Authors:** Ali Akbar Hussaini, Dilber Esra Yıldız, Filiz Boran, Murat Yıldırım

**Affiliations:** a Department of Biotechnology, Faculty of Science, Selcuk University 42130 Konya Türkiye; b Department of Physics, Faculty of Engineering and Natural Sciences, Hitit University 19030 Corum Türkiye desrayildiz@hitit.edu.tr; c Department of Chemical Engineering, Faculty of Engineering and Natural Sciences, Hitit University 19030 Corum Türkiye

## Abstract

In this study, Schottky photodetectors based on C-MWCNT/n-Si and Cryo-MWCNT/n-Si heterojunctions were fabricated and systematically investigated to evaluate the effect of cryogenic treatment on their electrical and optoelectronic properties. Initially, multi-walled carbon nanotubes (MWCNTs) were subjected to cryogenic treatment to produce Cryo-MWCNT, aiming to enhance their structural and surface characteristics. The modified nanotubes were characterized using Brunauer–Emmett–Teller (BET) surface area analysis, X-ray diffraction (XRD), Raman spectroscopy, scanning electron microscopy (SEM), and transmission electron microscopy (TEM) to verify the structural and morphological changes induced by the treatment. The photodetection properties were examined under illumination intensities ranging from 20 to 100 mW cm^−2^, where the Cryo-MWCNT/n-Si photodetector showed significantly enhanced photocurrent, photosensitivity, responsivity, and detectivity, along with reduced noise equivalent power (NEP). At an illumination intensity of 20 mW cm^−2^, the Cryo-MWCNT photodetector exhibited a responsivity of 0.1630 A W^−1^, a specific detectivity of 3.79 × 10^10^ Jones, and an NEP of 2.34 × 10^−12^ WHz^−1/2^. Broadband spectral measurements in the 351–1600 nm range under self-powered operation (0 V bias) confirmed the wide spectral sensitivity of the devices. At 900 nm, cryogenic treatment significantly enhanced the photodetector performance, yielding ∼20× higher photocurrent, responsivity, and EQE, ∼11.6× higher detectivity, and a ∼91% reduction in NEP compared to the untreated device. These results demonstrate that cryogenic treatment of MWCNTs greatly enhances photocarrier generation and transport, highlighting the Cryo-MWCNT/n-Si heterojunction as a strong contender for high-performance, broadband, self-powered photodetector applications.

## Abstract 2

In this study, Schottky photodetectors based on C-MWCNT/n-Si and Cryo-MWCNT/n-Si heterojunctions were fabricated and systematically investigated to evaluate the effect of cryogenic treatment on their electrical and optoelectronic properties.

## Graphical abstract

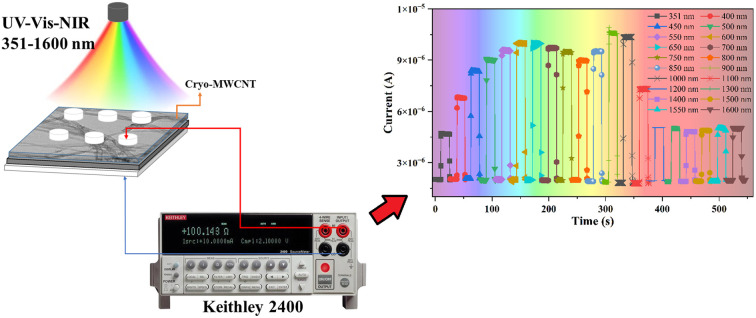

## 1. Introduction

Photodetectors (PDs) are key optoelectronic devices that convert incident light into electrical signals, making them essential for numerous technological and scientific applications.^1^ As functional devices, they transform elusive incident photons into quantifiable electrical outputs, making them essential for a wide range of technological and scientific applications.^2^ They are widely used in various application areas such as flame detection, optical communication, environmental monitoring, imaging systems, night vision, material identification, early tumor detection, health monitoring, and astronomical observations.^3–10^ In particular, photodetectors play a crucial role across both civilian and military domains: ultraviolet (UV) photodetectors are commonly employed in environmental monitoring, space science, and defense-related surveillance; visible (Vis) photodetectors are widely used in optical imaging, electronic vision systems, and solar energy harvesting; while infrared (IR) photodetectors are applied in optical communication, night vision technologies, remote sensing and control, analytical instrumentation, satellite-based Earth observation, and fire alarm systems.^11^ Traditionally, photodetectors are based on crystalline inorganic semiconductors fabricated on rigid wafer substrates. Among these, silicon-based photodetectors are extensively employed for visible light detection due to their high performance, low fabrication cost, and compatibility with established electronic technologies. For infrared (IR) detection, lower bandgap semiconductor materials such as InGaAs and HgCdTe are commonly utilized in applications including night vision, security systems, and biomedical imaging.^12^

Organic semiconducting materials possess promising optoelectronic properties and have been extensively utilized in low-cost photodetection technologies. Their strong absorption in the near-infrared (NIR) region, high absorption coefficients, lightweight nature, simple solution-based processing, and tunable optoelectronic characteristics make them highly attractive for next-generation photodetectors. These advantages provide significant potential to overcome several technical limitations associated with conventional inorganic-based photodetectors.^13–15^ These characteristics make them particularly attractive for emerging applications such as image sensing, wearable electronics, health monitoring, and distance measurement.^16,17^ Organic photodetectors are gradually transitioning from laboratory research to practical implementations. Their distinct advantages stem from employing organic semiconductors—π-conjugated molecules or polymers—which allow the creation of thin, lightweight, and flexible devices, addressing the rigidity limitations of conventional inorganic detectors. Moreover, through molecular design and optimized device architectures, OPDs can be tuned for either narrowband or broadband photodetection spanning the ultraviolet (UV), visible, and near-infrared (NIR) regions.^18^

MWCNTs are carbon-based materials known for their exceptional physical, chemical, and mechanical properties, as well as their large surface area.^19^ Commercially produced MWCNTs are naturally intertwined. However, their surface area can be increased by separating them using cutting processes.^20^ Current methods of cutting (chemical treatments, ultrasonic or mechanical milling) often damage the nanotubes, require long processing times and generate hazardous waste. Therefore, simpler and more scalable methods are needed. The reverse-principle thermal-stress method developed by Yirmişbeşoğlu *et al.*, which is based on rapid cooling and heating cycles under cryogenic conditions, offers a simple and industrially applicable approach to the functionalization of MWCNTs.^21^

In this study, MWCNTs were functionalized by applying thermal stress through rapid cooling followed by sudden heating under cryogenic conditions. The structural and surface changes resulting from this treatment were systematically examined using BET surface area analysis, XRD, Raman spectroscopy, SEM, and CTEM. In addition to structural characterization, the photodetection performance of both untreated and cryogenically treated MWCNTs was investigated under solar illumination as well as under different UV, Vis, and NIR wavelengths. This approach provides a novel strategy for enhancing the optoelectronic properties of MWCNT-based photodetectors through cryogenic thermal treatment.

## 2. Experimental details

### 2.1. Material

The commercial multi-walled carbon nanotubes (MWCNTs: outer diameter 10 ± 1 nm, inner diameter 4.5 ± 0.5 nm, length 3–6 µm) used in this study were obtained from Sigma-Aldrich. The liquid nitrogen used to create cryogenic conditions was obtained from HÜBTUAM.

### 2.2. Modification of MWCNTs

C-MWCNTs were functionalized *via* a thermal stress approach involving rapid cooling in liquid nitrogen followed by reheating to ambient temperature, as detailed in our previous study.^21^ In that work, the process parameters were systematically optimized by varying the number of cycles, the residence time in liquid nitrogen, and the holding time at room temperature. The optimum conditions were determined to be 2 cycles, each consisting of immersion in liquid nitrogen for 20 min followed by exposure to room temperature for 5 min, which produced MWCNTs with the highest surface area and improved structural properties. Therefore, in the present study, MWCNT functionalization was carried out under these previously optimized conditions. Briefly, the MWCNTs were placed in falcon tubes and immersed in liquid nitrogen (∼−196 °C) for 20 min. They were then transferred rapidly to room temperature for 5 min, and this procedure was repeated for two cycles. The resulting samples were then used for further characterization and investigation of their additional properties.

### 2.3. Device fabrication

Phosphorus-doped n-type silicon wafers (Wafer World, USA) with a resistivity of 1–20 Ω cm and a thickness of 500–550 µm were diced into 10 × 10 mm^2^ pieces. The samples were cleaned using the standard RCA process,^22^ followed by treatment with a diluted HF solution to remove native oxide and surface impurities. A 120 nm thick aluminum layer was then deposited on the backside of the silicon *via* physical vapor deposition (PVD) to establish the ohmic contact. To enhance ohmic contact formation, the samples were annealed at 450 °C for 5 minutes in a nitrogen atmosphere. Subsequently, 50 µL of each prepared C-MWCNT and Cryo-MWCNT solution (20 mg mL^−1^) was separately applied to the front surface of the n-Si substrates and evenly distributed by spin coating at 2000 rpm for 40 seconds. The coated samples were then left to dry. Finally, 120 nm thick Al top contacts were deposited onto the coated surface by PVD using a shadow mask, resulting in an effective contact area of 7.85 × 10^−3^ cm^2^.

**Fig. 1:**
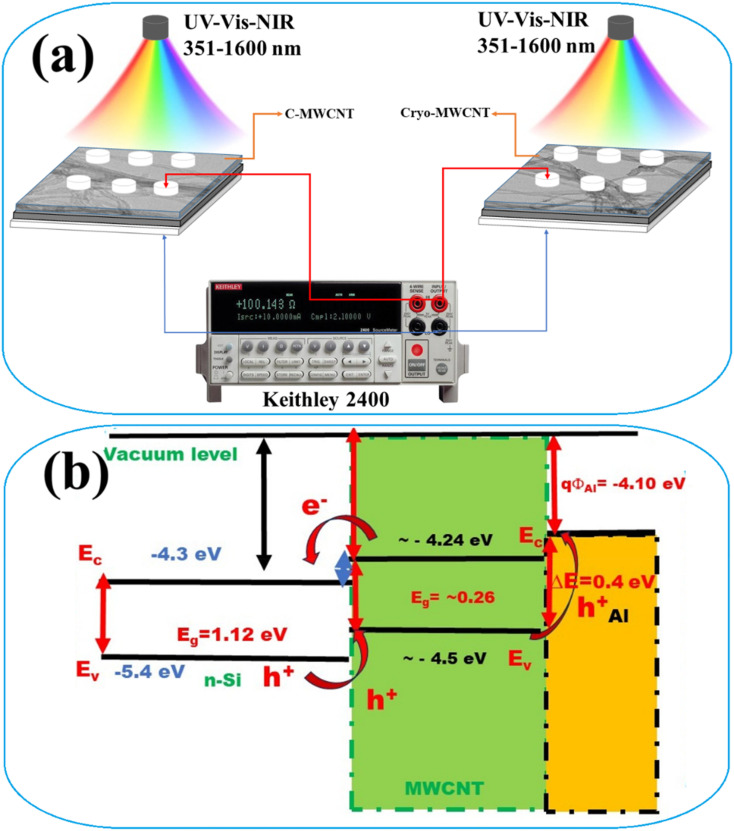
(a) Schematic illustration of the C-MWCNT/n-Si and Cryo-MWCNT/n-Si devices and (b) the energy band diagram of Al/MWCNT/n-Si device.

The organic materials used in this study were Commercial-MWCNT and Cryo-MWCNT, and the corresponding devices—C-MWCNT/n-Si and Cryo-MWCNT/n-Si—were successfully fabricated. A schematic representation of the fabricated device structure is presented in Fig. 1a. As seen Fig. 1b, the conduction band minimum (*E*_c_ = −4.3 eV) and valence band maximum (*E*_v_ = −5.4 eV) of n-Si define the carrier generation region and n-Si has a bandgap of 1.12 eV. As it well known, n-Si generates electron–hole pairs under illumination this region. Also, MWCNT layer exhibits a very narrow bandgap^23^ and the work function of MWCNTs was referred to in the previous report.^24^ The work function of MWCNT is −4.5 eV (ref. 24 and 25) and the HOMO level of MWCNT is −5.1 eV.^26^ This alignment allows holes (h^+^) generated in Si to transfer efficiently into the MWCNT valence band, while electrons face a barrier. In additionally, the work function of Al is ∼4.1 eV. Photo-excited holes in n-Si drift toward the interface and are injected into the MWCNT layer to Al due to favorable band alignment.^27,28^ Similarly, electrons (e^−^) remain confined in Si, reducing recombination at the interface. This selective transport enhances photocurrent and improves device rectification.^27,29^ The energy band diagram of the Al/MWCNT/n-Si heterostructure reveals that the narrow bandgap of MWCNTs provides a favorable pathway for hole transport from the valence band of n-Si into the carbon nanotube layer. Under illumination, photo-generated holes in silicon are efficiently injected into the MWCNT valence band, while electrons encounter a barrier, thereby suppressing recombination and enhancing rectification. The Al electrode forms a contact with MWCNTs, facilitating hole extraction to the external circuit. This selective transport mechanism explains the strong photocurrent observed in CNT/Si heterojunction devices and aligns with previous reports on CNT/Si solar cells and photodetectors, where efficient hole transfer and interface stability were demonstrated.^27,28^

### 2.4. Characterization of commercial and modified MWCNTs

XRD analysis was performed using a Rigaku DMAX IIIC diffractometer with CuKα radiation (*λ* = 1.541871 Å) at a scan rate of 2° min^−1^. Raman spectra were recorded using a Renishaw in *Via* Raman microscope with a 532 nm laser in the range of 0–3500 cm^−1^ at room temperature. Surface area measurements were carried out by the BET method using N_2_ adsorption at 77 K on a Quantachrome IQ-Chemi system. Before analysis, the samples were degassed under vacuum at 200 °C for 24 h. Morphological characterization of the MWCNTs was conducted using a FEI Tecnai G2 Spirit Biotwin CTEM microscope. For TEM analysis, powdered samples were ultrasonically dispersed in ethanol for 1 h, deposited onto a grid, and dried overnight. The surface morphology of the samples was examined using scanning electron microscopy (SEM, ZEISS EVO LS10, Carl Zeiss AG, Germany). Electrical and optoelectronic properties were evaluated using a Keithley 2400 SourceMeter in conjunction with an FY-7000 solar simulator. *I*–*V* measurements were recorded under illumination intensities of 20, 40, 60, 80, and 100 mW cm^−2^ to assess device photoresponse at varying power levels. Spectral response was measured using 20 narrowband visible-NIR band-pass filters from Thorlabs GmbH (Germany), each with an average FWHM of ∼10 nm, enabling accurate wavelength selection. Measurements were performed across 351–1600 nm at a fixed intensity of 20 mW cm^−2^ to analyze wavelength-dependent optoelectronic behavior.

## 3. Results and discussion

### 3.1. Structural and morphological characterization

The functionalization of commercial MWCNTs by thermal stress treatment under cryogenic conditions resulted in the removal of amorphous carbon and other impurities, which increased the BET surface area from 252.439 m^2^ g^−1^ to 324.312 m^2^ g^−1^. This enhancement can be attributed to the elimination of amorphous carbon layers and impurities that previously blocked the surface, thereby exposing additional active sites and providing a larger accessible area.^30^

**Fig. 2:**
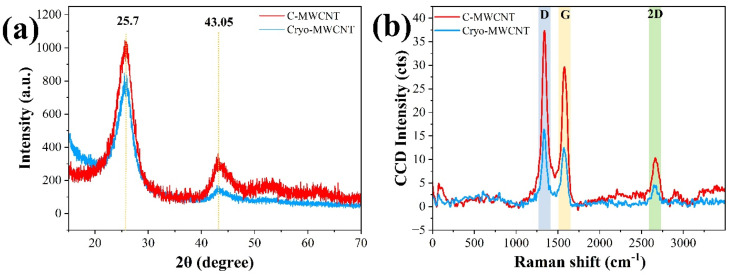
(a) XRD patterns and (b) Raman spectra of commercial and modified MWCNTs.

The XRD patterns in Fig. 2a for both commercial and modified MWCNTs show two prominent diffraction peaks at 2*θ* = 25.7° and 43.05°, assigned to the (0 0 2) and (1 0 0) planes of graphitic carbon, respectively (JCPDS card no. 75-1621) (Fig. 2a).^31,32^ Comparison of the two patterns reveals that these peak intensities decrease after liquid nitrogen treatment. This reduction suggests that the modification process significantly affects the structural properties of the material.

In Fig. 2b, the G, D, and 2D bands of the samples modified in the liquid nitrogen environment appear at positions similar to those of the commercial MWCNTs, with only slight shifts observed. This behavior can be associated with the functionalization of the commercial MWCNTs induced by thermal stress. In the modified sample, the CCD intensity was found to decrease, which can be attributed to the functionalization process occurring during the thermal stress treatment. Raman spectral parameters are also listed in Table 1. The ratio of the two peak intensities (*I*_D_/*I*_G_) is commonly used to evaluate the structural quality of carbon materials. As the *I*_D_/*I*_G_ ratio approaches zero, it indicates a more ordered arrangement of carbon atoms.^33^ An increase in the *I*_D_/*I*_G_ ratio is typically associated with the introduction of defects in the carbon lattice, often caused by the displacement or sputtering of carbon atoms, while a decrease in this ratio generally indicates partial structural healing or recovery. In this work, the *I*_D_/*I*_G_ values of both the commercial and modified MWCNTs were found to be nearly identical. This suggests that the level of structural defects in the modified MWCNTs remains comparable to that of the commercial samples.

**Table 1:** Raman spectral parameters of commercial and modified MWCNTs

Samples	D band (cm^−1^)	*I*_D_	G band (cm^−1^)	*I*_G_	2D band (cm^−1^)	*I*_2D_	*I*_D_/*I*_G_
C-MWCNT	1337.24	37.48	1577.22	29.79	2665.22	10.40	1.26
Cryo-MWCNT	1333.10	16.45	1571.34	12.65	2648.32	4.62	1.30

**Fig. 3:**
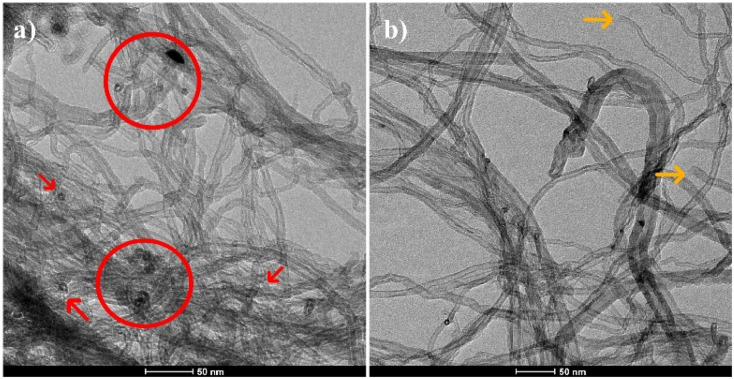
CTEM micrographs of (a) commercial MWCNTs and (b) modified MWCNTs samples [scale bar: 50 nm].

CTEM analysis was performed to examine the surface characteristics of both commercial and modified MWCNT samples. The obtained micrographs provided important insights into the structural changes induced by thermal shearing during the modification process. As shown in Fig. 3a, the CTEM image of the commercial MWCNTs reveals nanotubes predominantly terminated with closed end caps. Also, Fig. 3b shows that the modified MWCNTs possess rough and open tube ends. Furthermore, the modification treatment reduced the degree of nanotube entanglement. TEM observations also indicated that the thermal stress applied during the modification process promoted the opening and functionalization of graphitic end caps, which contributed to the shortening of the initially long MWCNTs.

**Fig. 4:**
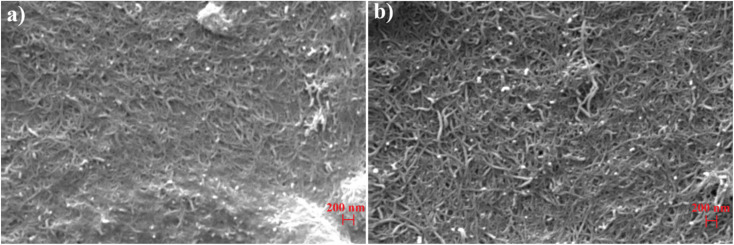
SEM micrographs of (a) commercial MWCNTs and (b) modified MWCNTs.

Fig. 4 shows the SEM micrographs of the pristine MWCNTs (Fig. 4a) and cryogenically treated MWCNTs (Fig. 4b). Both samples exhibit a randomly oriented and highly interconnected network of entangled nanotubes, indicating the preservation of the characteristic fibrous morphology after cryogenic treatment. No noticeable structural damage, such as nanotube breakage or collapse, is observed in the cryogenically treated sample. Compared with the pristine MWCNTs, the cryogenically treated MWCNTs appear to exhibit a more homogeneous distribution and slightly reduced agglomeration, resulting in a more open and uniform network structure. These observations suggest that the cryogenic treatment does not significantly alter the overall morphology of the MWCNTs but may influence their dispersion and intertube arrangement.

### 3.2. Electrical and optoelectrical measurements

**Fig. 5:**
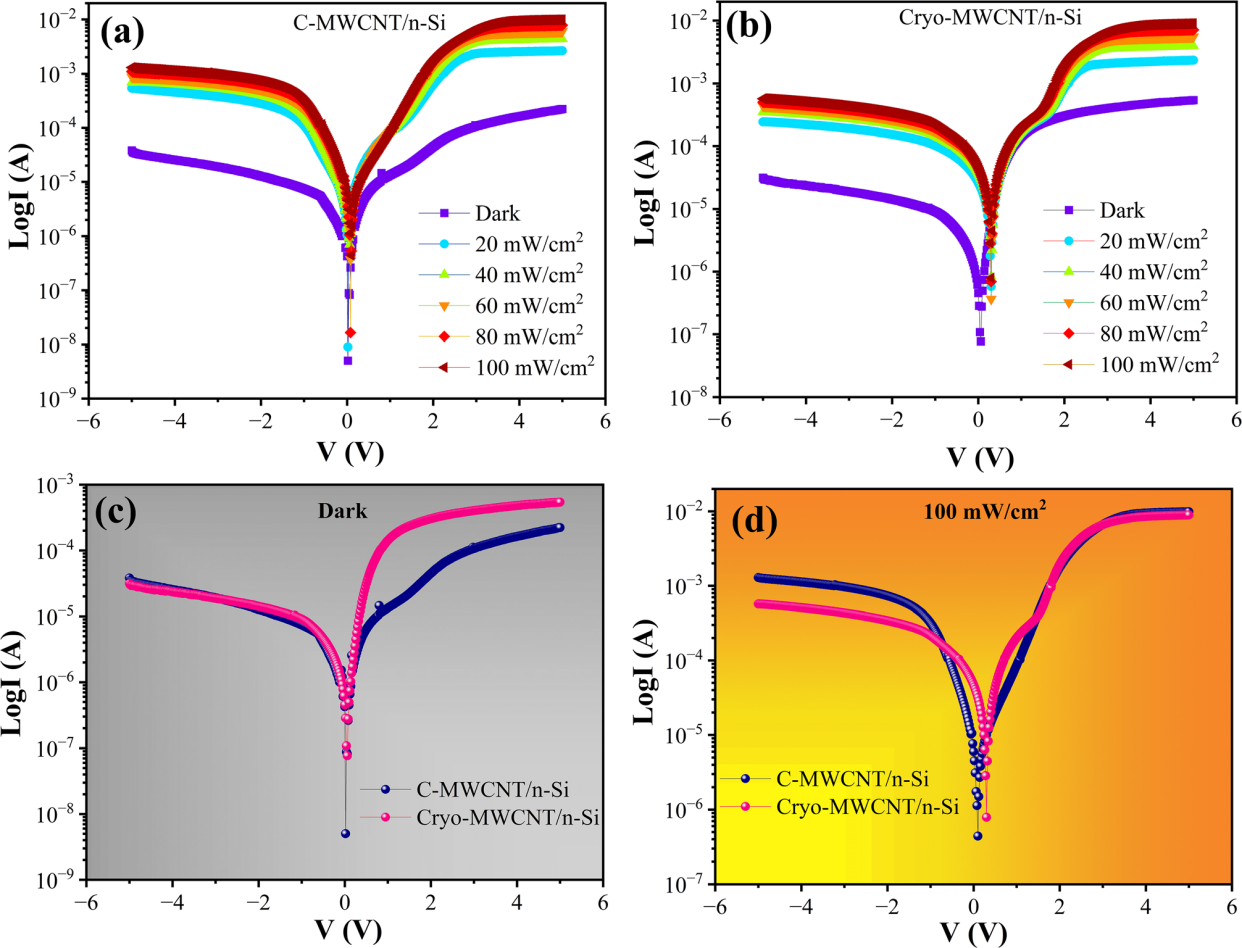
*I*–*V* plots of (a) C-MWCNT/n-Si and (b) Cryo-MWCNT/n-Si devices; comparison of the *I*–*V* plot of the devices measured at (c) dark and (d) 100 mW cm^−2^.

The current–voltage (*I*–*V*) behavior of the fabricated devices was examined over a voltage range of −5 V to +5 V under both dark and illuminated conditions. To evaluate their photoresponse, the illumination intensity was varied systematically at 20, 40, 60, 80, and 100 mW cm^−2^. As shown in Fig. 5a and b, the photocurrent steadily increases with rising light intensity for both C-MWCNT/n-Si and Cryo-MWCNT/n-Si devices. This enhancement is observed in both forward and reverse bias regions, indicating efficient generation and transport of charge carriers. Under illumination, incoming photons create electron–hole pairs within the semiconductor, which are then separated by the built-in electric field at the metal–semiconductor junction. In reverse bias, the depletion region widens and the electric field becomes stronger, facilitating more effective separation and collection of these carriers, thereby significantly increasing the photocurrent. In forward bias, the barrier at the junction is lowered, allowing more photogenerated carriers to contribute to conduction. Furthermore, Fig. 5c and d compare the *I*–*V* characteristics of C-MWCNT/n-Si and Cryo-MWCNT/n-Si devices under dark conditions and at an illumination intensity of 100 mW cm^−2^. A clear rise in current under illumination is observed, demonstrating strong photoresponse and confirming the typical photodiode behavior of the MWCNT/Si heterojunction devices. As shown in Fig. 5c and d, the photocurrent under reverse bias is markedly higher than that under forward bias for both C-MWCNT/n-Si and Cryo-MWCNT/n-Si junctions. This trend has been consistently reported in the literature for MWCNT/Si heterojunction photodetectors, where reverse bias enhances the depletion-region electric field and thereby accelerates the separation of photogenerated electron–hole pairs while suppressing recombination. For instance, Ismail *et al.* demonstrated that a spray-deposited MWCNT/Si heterojunction exhibited a photocurrent-to-dark-current ratio of ∼1.16 × 10^3^ at 8 V reverse bias under 100 mW cm^−2^ illumination.^29^ Similarly, Pelella *et al.* reported tunable photocurrent enhancement in CNT/Si_3_N_4_/Si structures under reverse bias.^34^ Theoretical and experimental studies further corroborate this mechanism: Marsh *et al.* directly observed that photocurrent generation under reverse bias arises from field-assisted dissociation of Coulomb-bound charge pairs,^35^ while Fujioka *et al.* showed that reverse bias broadens the depletion region and facilitates carrier injection in LaMnO_3_/SrTiO_3_ heterojunctions.^36^ In CNT/Si systems, enhanced carrier extraction under reverse bias has been documented by An *et al.*,^37^ Salvato *et al.*,^38^ and others, with responsivities up to 0.10 A W^−1^ and photocurrent gains exceeding 30-fold compared to photovoltaic mode operation. Importantly, this observation is consistent with the proposed hole transport mechanism, since reverse bias promotes hole injection and transport across the CNT/Si interface, thereby reinforcing the device's photoresponse. This mechanism is further supported by ultrafast pump–probe spectroscopy: Ponzoni *et al.* tracked the transfer of photogenerated holes from the Si depletion region into CNT layers, highlighting the active role of CNTs in interfacial transport.^39^ More recently, Zhang *et al.* confirmed *via* first-principles calculations that reverse bias enhances carrier extraction and photocurrent,^40^ while Salvato *et al.* reported both fast and high photoresponse in SWCNT/n-Si Schottky photodetectors, consistent with hole transport across the junction.^41^

**Fig. 6:**
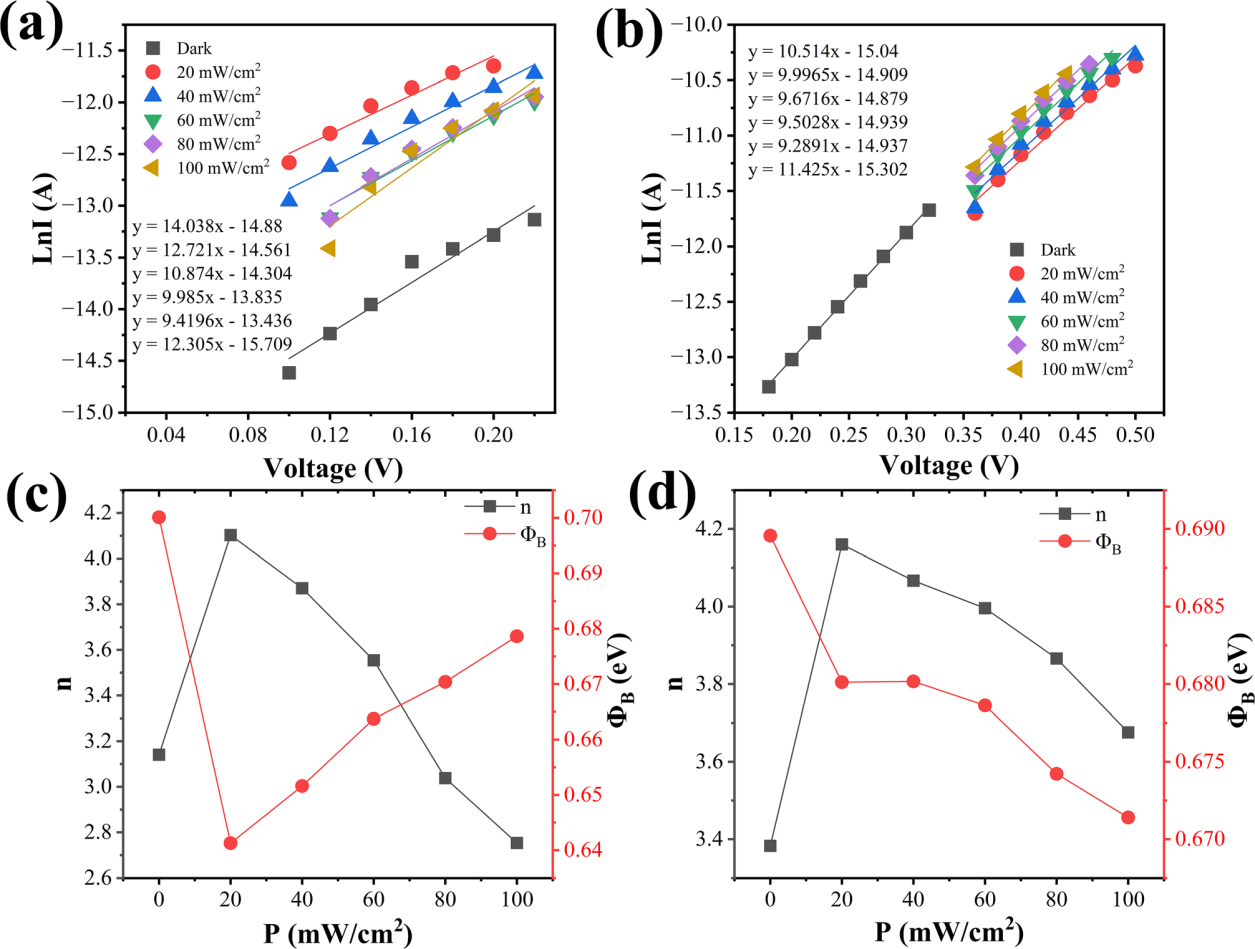
Ln *I*–*V* plots of (a) C-MWCNT/n-Si and (b) Cryo-MWCNT/n-Si devices; n-*Φ*_B_ plots of (c) C-MWCNT/n-Si and (d) Cryo-MWCNT/n-Si devices.

Fig. 6a–d illustrate the electrical characteristics and parameter extraction of the device under dark and illuminated conditions at different light intensities. In Fig. 6a and b, the semi-logarithmic plots of current (ln *I*) *versus* applied voltage show a clear linear region, indicating that the device follows an exponential current–voltage behavior consistent with diode conduction. As the illumination power increases from dark to 100 mW cm^−2^, the current increases and the curves shift upward, demonstrating enhanced carrier generation due to incident. The linear fitting equations shown in the plots are used to extract key diode parameters. In C-MWCNT/n-Si device, the slopes obtained from the linear fitting of the ln(*I*) *versus* voltage plots represent the exponential growth rate of the current and vary with illumination intensity. The slope value of 12.30 corresponds to the dark condition, while the slopes 9.41, 9.98, 10.87, 12.72, and 14.03 correspond to illumination intensities of 20, 40, 60, 80, and 100 mW cm^−2^, respectively. The change in slope with increasing light intensity indicates that illumination affects the charge transport mechanism in the device. Specifically, the increase in slope at higher illumination levels suggests enhanced carrier generation and improved current conduction due to the increased number of photogenerated charge carriers within the semiconductor.

For the Cryo-MWCNT/n-Si device, the slopes extracted from the linear fitting of the ln(*I*) *versus* voltage curves also vary with illumination intensity. The slope value of 11.425 corresponds to the dark condition, while the slopes 9.289, 9.503, 9.672, 9.996, 10.514, and correspond to illumination intensities of 20, 40, 60, 80, and 100 mW cm^−2^, respectively. As the illumination intensity increases from 20 to 100 mW cm^−2^, the slopes change from 10.514 to 9.289. The gradual decrease in slope indicates that the current increases more rapidly with light exposure due to the higher number of photogenerated carriers. This behavior suggests improved conductivity and enhanced carrier transport in the device under illumination.

Thermionic emission is the process by which charge carriers are released from a heated surface. In metal–semiconductor rectifier contacts, it occurs when carriers on either the metal or semiconductor side gain enough thermal energy to overcome the potential barrier at the interface. For metal contacts with an n-type semiconductor, electrons are the carriers responsible for this process, whereas for metal contacts with a p-type semiconductor, the emission is carried out by holes.^42,43^ The thermionic emission (TE) model was employed to determine the ideality factor (*n*) and the Schottky barrier height (*Φ*_B_). Fig. 6c and d illustrate the dependence of these parameters on illumination intensity. In general, the *n* value decreases as the optical power increases, which implies enhanced charge transport and reduced carrier recombination under stronger illumination. Meanwhile, the Schottky barrier height exhibits only minor variations with increasing light intensity, suggesting that illumination subtly modifies the effective barrier at the metal–semiconductor interface. These results confirm that intensity significantly affects the transport properties and electrical performance of the device.

Although the device is referred to as a Schottky contact-based photodetector in this manuscript, previous studies have shown that the MWCNT/n-Si interface exhibits characteristics that are more complex than those of a conventional Schottky junction. Behnam *et al.* determined the CNT film work function (∼4.5–4.7 eV) *via C*–*V* measurements and demonstrated that thermionic emission dominates at high temperature, while tunneling prevails at low temperature, thereby directly supporting the Schottky regime and thermionic emission argument.^44^ In contrast, Tzolov *et al.* (2004) reported a large type-I band offset and rectification ratio (on/off ∼10^5^), showing that the CNT/Si interface can also function as a heterojunction with depletion-region formation and p–n-like carrier separation.^45^ Kuo *et al.* further consolidated this duality by observing Schottky diode behavior, space-charge-limited current, and heterodimensional field emission within the same CNT/Si device, underscoring the coexistence of multiple regimes.^46^ Jung *et al.* provided strong evidence for heterojunction-like hole injection and carrier separation in efficient SWNT/n-Si solar cells, where transport was governed by diffusion rather than Schottky barrier emission, with minority carrier lifetimes of ∼34 µs.^47^ Chen *et al.* also demonstrated that in CNT transistors, the transition from thermionic emission to ohmic tunneling contacts is controlled not by work function alone but by channel doping, confirming that the metallic or semiconducting character of the CNT film dictates the dominant transport mechanism.^48^ Taken together, these reports indicate that the MWCNT/n-Si junction may exhibit both Schottky-like and heterojunction-like behavior depending on the CNT electronic character, doping state, and measurement conditions. A clearer description of this aspect would help explain the higher photocurrent under reverse bias and provide better insight into the dominant carrier transport mechanism at the CNT/Si interface.

**Fig. 7:**
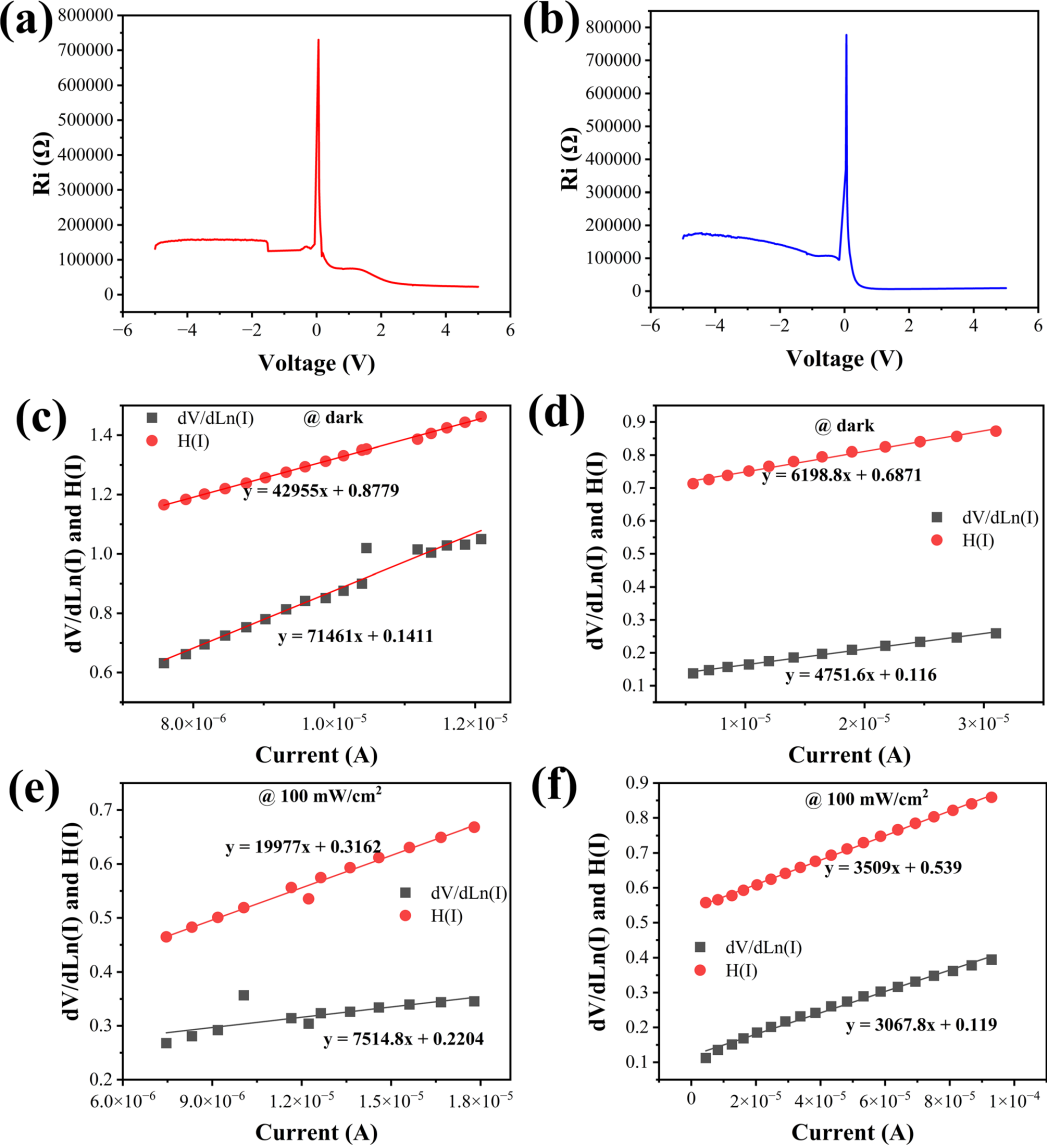
*R*_i_–*V* plots of (a) C-MWCNT/n-Si and (b) Cryo-MWCNT/n-Si devices; Cheung plots of (c) C-MWCNT/n-Si and (d) Cryo-MWCNT/n-Si devices at dark; Cheung plots of the (e) C-MWCNT/n-Si and (f) Cryo-MWCNT/n-Si devices at 100 mW cm^−2^.

Fig. 7a and b show the variation of the series resistance (*R*_i_) as a function of applied voltage for the C-MWCNT/n-Si and Cryo-MWCNT/n-Si devices, respectively. In both devices, *R*_i_ exhibits a relatively high and nearly constant value in the reverse bias region, followed by a sharp peak near 0 V, which corresponds to the transition region between reverse and forward bias. This pronounced peak indicates the strong influence of the junction barrier and interface states on carrier transport. After this peak, the resistance decreases significantly in the forward bias region, reflecting enhanced carrier injection and improved conduction through the device.

Fig. 7c and d present the Cheung's method analysis under dark conditions for C-MWCNT/n-Si and Cryo-MWCNT/n-Si devices, where the functions d*V*/d ln(*I*) and *H*(*I*) are plotted as a function of current. The slopes of these lines are used to determine the series resistance, while the intercepts are related to the *n* and *Φ*_B_ values. For the C-MWCNT/n-Si device, the slopes obtained from the d*V*/d ln(*I*) plots are 71 461 Ω under dark conditions (Fig. 7c) and 7514.8 Ω under illumination at 100 mW cm^−2^ (Fig. 7e). This large reduction in resistance under illumination indicates that photogenerated carriers significantly enhance the conductivity of the device.

For the Cryo-MWCNT/n-Si device, the slopes of the d*V*/d ln(*I*) plots are 4751.6 Ω in the dark (Fig. 7d) and 3067.8 Ω under 100 mW cm^−2^ illumination (Fig. 7f). Compared with the C-MWCNT/n-Si device, the Cryo-MWCNT/n-Si device exhibits much lower series resistance in both dark and illuminated conditions.

**Fig. 8:**
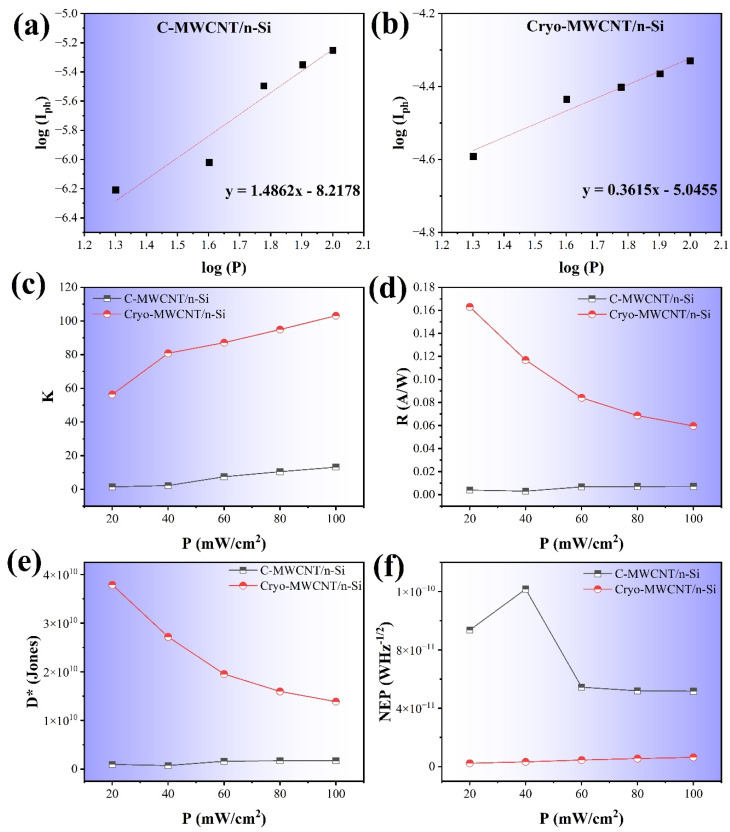
Log(*P*)–log(*I*_ph_) plots of the (a) C-MWCNT/n-Si and (b) Cryo-MWCNT/n-Si devices; (c) photosensitivity, (d) responsivity, (e) detectivity, and (f) NEP plots of the devices.

Fig. 8a and b show the logarithmic dependence of photocurrent on illumination power for the C-MWCNT/n-Si and Cryo-MWCNT/n-Si devices. The photoresponse characteristics of these devices can be evaluated using the empirical power-law relation:

1 *I*_ph_ = *αP*^*m*^

where *α* represents a proportionality constant, while *m* indicates the level of linearity between the photocurrent and the incident optical power. The value of *m* is determined from the slope of the log(*I*_ph_) *versus* log(*P*) plots. The C-MWCNT/n-Si and Cryo-MWCNT/n-Si devices exhibit *m* values of 1.4862 and 0.3615, respectively, indicating distinctly different photoresponse behaviors. The super-linear dependence observed for the C-MWCNT/n-Si device (*m* > 1) suggests the presence of photoconductive gain and trap-filling processes, whereby the photocurrent increases more rapidly than the incident optical power.^49,50^ In contrast, the Cryo-MWCNT/n-Si device exhibits a sub-linear response (*m* < 1), indicating that trap states play a significant role in the photocurrent generation process. Such sub-linear behavior is commonly attributed to trap-assisted carrier dynamics and photogating effects, where photogenerated carriers become temporarily trapped and modulate the conductivity of the device.^51^ Although the Cryo-MWCNT/n-Si device shows a lower *m* value, its superior photodetector performance in terms of other key parameters indicates that cryogenic treatment enhances charge separation and carrier collection efficiency. Therefore, the reduced *m* value reflects a change in the dominant photoresponse mechanism rather than a deterioration in device performance, suggesting that trap-mediated processes contribute to the improved photodetection characteristics of the Cryo-MWCNT/n-Si device.

Several parameters are used to evaluate the performance of a photodetector, including photocurrent, photosensitivity (*K*), responsivity (*R*), noise equivalent power (NEP), and detectivity (D*). These parameters can be determined using the following equations.^52–55^

2 *I*_ph_ = *I*_light_ − *I*_dark_

3
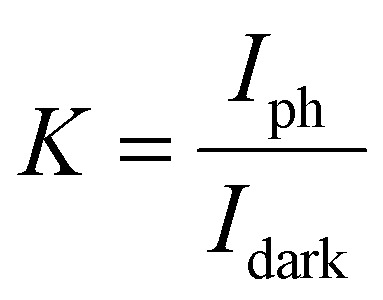
4
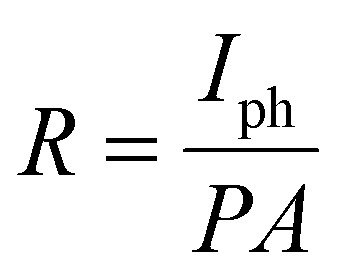
5
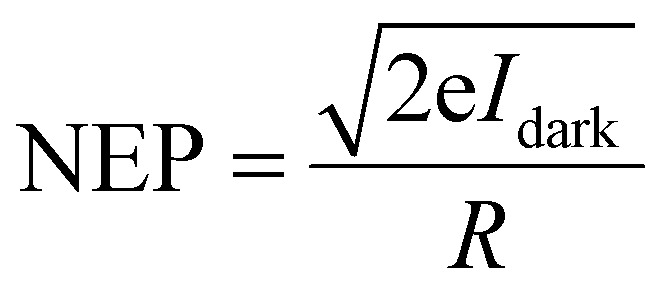
6
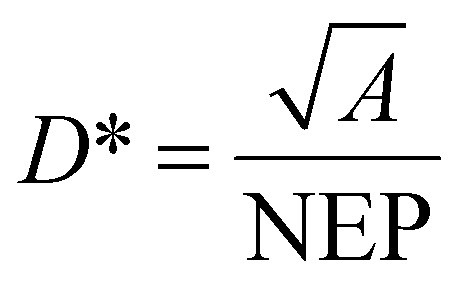
In these equations, (*P*) represents the incident optical power density, while (*A*) corresponds to the effective area of the photodetector. Responsivity (*R*) describes the efficiency with which the device converts incident light into an electrical signal. Photosensitivity (*K*) is defined as the ratio of photocurrent to dark current and indicates the device's sensitivity to illumination. The noise-equivalent power (NEP) is the minimum optical power that can be distinguished from the noise background, making it a key parameter for evaluating detector sensitivity. Specific detectivity (D*) characterizes the capability of the photodetector to detect weak optical signals and is normalized with respect to the detector area and measurement bandwidth.^56^

Table 2 and Fig. 8c–f show a clear performance difference between the C-MWCNT/n-Si and Cryo-MWCNT/n-Si photodetectors under illumination intensities from 20 to 100 mW cm^−2^. Overall, the Cryo-MWCNT device demonstrates significantly superior photoresponse characteristics. First, the photocurrent of the Cryo-MWCNT device is higher than that of the C-MWCNT device at all illumination powers. For example, at 20 mW cm^−2^, the photocurrent increases from 6.18 × 10^−7^ A for C-MWCNT to 2.56 × 10^−5^ A for Cryo-MWCNT. Similarly, at 100 mW cm^−2^, it rises from 5.60 × 10^−6^ A to 4.68 × 10^−5^ A. This indicates that the Cryo-treated interlayer significantly enhances carrier generation and collection. A similar trend is observed for photosensitivity. The C-MWCNT device shows values ranging from 1.45 to 13.18, whereas the Cryo-MWCNT device exhibits much larger values from 56.35 to 103.11, confirming the stronger light-response capability of the Cryo-MWCNT structure.

**Table 2:** Photodetector parameters under various solar light intensities

Interlayer	Power (mW cm^−2^)	Photocurrent (*A*)	Photosensitivity	Responsivity (A W^−1^)	Detectivity (Jones)	NEP (WHz^−1/2^)
C-MWCNT	20	6.18 × 10^−7^	1.45	0.0039	9.46 × 10^8^	9.37 × 10^−11^
	40	9.51 × 10^−7^	2.24	0.0030	7.28 × 10^8^	1.22 × 10^−10^
	60	3.19 × 10^−6^	7.51	0.0068	1.63 × 10^8^	5.45 × 10^−11^
	80	4.45 × 10^−6^	10.48	0.0071	1.70 × 10^9^	5.20 × 10^−11^
	100	5.60 × 10^−6^	13.18	0.0071	1.71 × 10^9^	5.17 × 10^−11^
Cryo-MWCNT	20	2.56 × 10^−5^	56.35	0.1630	3.79 × 10^10^	2.34 × 10^−12^
	40	3.67 × 10^−5^	80.82	0.1169	2.72 × 10^10^	3.26 × 10^−12^
	60	3.96 × 10^−5^	87.17	0.0841	1.95 × 10^10^	4.54 × 10^−12^
	80	4.31 × 10^−5^	94.95	0.0687	1.60 × 10^10^	5.55 × 10^−12^
	100	4.68 × 10^−5^	103.11	0.0597	1.39 × 10^10^	6.39 × 10^−12^

The responsivity (*R*) also shows a remarkable improvement. In the C-MWCNT device, responsivity varies between 0.0030 and 0.0071 A W^−1^, while in the Cryo-MWCNT device it ranges from 0.0597 to 0.163 A W^−1^. This indicates a much more efficient conversion of incident optical power into electrical current in the Cryo-MWCNT device. For detectivity (D*), the Cryo-MWCNT device again shows superior performance, reaching values on the order of 10^10^ Jones, whereas the C-MWCNT device remains around 10^8^–10^9^ Jones. Higher detectivity reflects a better ability to detect weak optical signals. Conversely, the NEP, which represents the minimum detectable optical power, is significantly lower for the Cryo-MWCNT device. The NEP decreases from approximately 10^−10^–10^−11^ WHz^−1/2^ for C-MWCNT to about 10^−12^–10^−12^ WHz^−1/2^ for Cryo-MWCNT, indicating improved sensitivity and reduced noise. Overall, the comparison demonstrates that the Cryo-MWCNT/n-Si photodetector exhibits substantially enhanced photocurrent, photosensitivity, responsivity, and detectivity, along with lower NEP, compared with the conventional C-MWCNT/n-Si device. These improvements can be attributed to the better electrical transport properties and improved interface quality introduced by the cryogenic treatment of the MWCNT interlayer, which facilitates more efficient photogenerated carrier separation and collection.

**Fig. 9:**
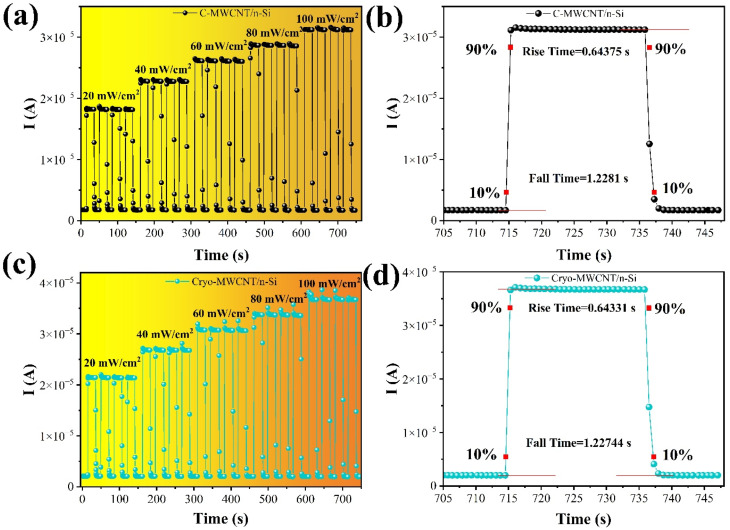
(a) *I*–*t* and (b) rise/fall time plots of the C-MWCNT/n-Si device; (c) *I*–*t* and (d) rise/fall time plots of the Cryo-MWCNT/n-Si device.

Fig. 9a and c present the time-dependent photoresponse characteristics of the C-MWCNT/n-Si and Cryo-MWCNT/n-Si photodetectors under periodically modulated illumination with light intensities varying from 20 to 100 mW cm^−2^. For both devices, the current rises immediately upon light exposure and rapidly returns to its baseline value once the illumination is turned off, confirming a stable, reproducible, and reversible ON/OFF switching behavior throughout repeated illumination cycles. This repeatability demonstrates the good operational stability and reliability of the fabricated photodetectors. Furthermore, the photocurrent increases systematically as the illumination intensity increases, indicating that both devices possess strong sensitivity to incident light power and efficient photogenerated carrier production.

A noticeable difference can be observed between the two devices, where the Cryo-MWCNT/n-Si photodetector consistently exhibits a higher photocurrent than the C-MWCNT/n-Si device at all investigated illumination intensities. This enhancement suggests that the cryogenic treatment of the MWCNT interlayer improves charge carrier generation, separation, and transport across the heterojunction interface. The improved electrical pathways and reduced carrier recombination resulting from cryogenic processing likely contribute to the superior photoresponse performance of the Cryo-MWCNT/n-Si structure. Consequently, the cryogenically treated device demonstrates enhanced light-harvesting capability and more efficient photocarrier extraction under illumination.

Fig. 9b and d illustrate the transient switching characteristics of the devices, which were analyzed to evaluate their response speed through rise and fall time measurements. The rise time is defined as the duration required for the photocurrent to increase from 10% to 90% of its peak value after illumination is applied, whereas the fall time corresponds to the time needed for the photocurrent to decrease from 90% to 10% after the light source is switched off. For the C-MWCNT/n-Si photodetector, the rise and fall times were determined to be 0.64375 s and 1.2281 s, respectively. In comparison, the Cryo-MWCNT/n-Si device exhibits rise and fall times of 0.64331 s and 1.22744 s, respectively.

The very small difference between the response times of the two devices indicates that both photodetectors possess fast, stable, and efficient photo-switching characteristics with minimal delay during illumination transitions. Although the response speeds remain nearly identical, the Cryo-MWCNT/n-Si device delivers a significantly larger photocurrent while preserving rapid switching behavior. This result demonstrates that cryogenic treatment enhances the photodetection efficiency without compromising the dynamic response characteristics of the device. Overall, the improved photocurrent generation combined with stable rise and fall times confirms the positive role of cryogenic treatment in optimizing the optoelectronic performance of the MWCNT/n-Si photodetector.

**Fig. 10:**
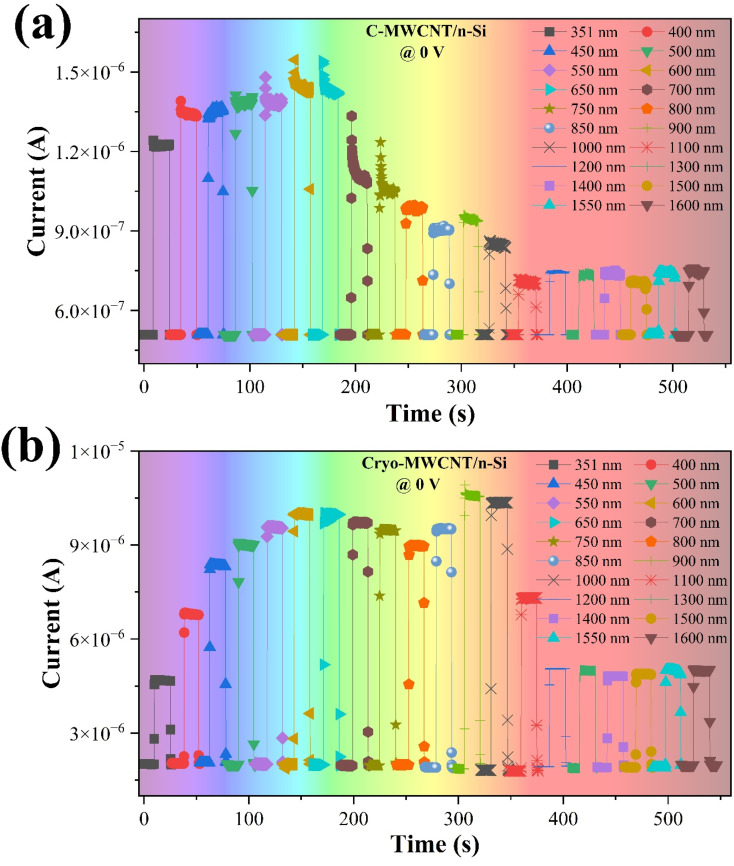
*I*–*t* plots of the (a) C-MWCNT/n-Si and (b) Cryo-MWCNT/n-Si devices.

Fig. 10a and b present the wavelength-dependent photocurrent responses of the C-MWCNT/n-Si and Cryo-MWCNT/n-Si photodetectors, respectively, measured under zero-bias (self-powered) operating conditions. The spectral measurements were carried out using a series of optical filters that enabled characterization across a broad wavelength range extending from the UV region through the visible spectrum and into the near-infrared (NIR) region, covering wavelengths from 351 to 1600 nm. During all measurements, the incident optical power density was kept constant at 20 mW cm^−2^ for each selected wavelength to ensure identical illumination conditions throughout the experiment. Maintaining a fixed optical power density eliminates variations associated with incident light intensity and allows an accurate comparison of the intrinsic spectral response behavior of the fabricated photodetectors over the investigated spectral range. The obtained results clearly demonstrate that both devices possess broadband photodetection capability, as indicated by the generation of measurable photocurrent signals across the entire UV-visible-NIR spectrum. The ability to respond over such a wide spectral range suggests that the heterojunction structures are capable of efficiently absorbing photons with different energies and converting them into electrical signals. The photoresponse observed in the UV region can be attributed to high-energy photon absorption near the surface of the device, while the strong response in the visible and near-infrared regions is mainly associated with the optical absorption characteristics of silicon combined with the conductive and light-interacting properties of the MWCNT interlayer. The broad spectral sensitivity obtained in these devices makes them highly promising for multifunctional photodetection applications, including environmental monitoring, optical communication, imaging systems, and broadband optoelectronic sensing technologies.

An important feature of the measurements is that all photocurrent responses were obtained under self-powered conditions without applying any external bias voltage. This confirms the photovoltaic behavior of the fabricated heterojunction photodetectors and indicates the presence of an effective built-in electric field at the MWCNT/n-Si interface. Under illumination, the generated electron–hole pairs are efficiently separated and transported by this internal electric field, enabling photocurrent generation even in the absence of an external power supply. Such self-powered operation is highly advantageous for practical applications because it significantly reduces energy consumption, simplifies device architecture, and enhances operational stability. The ability to achieve broadband photodetection under zero-bias conditions demonstrates the suitability of these devices for low-power and energy-efficient optoelectronic systems.

A comparison between Fig. 10a and b further reveals that the Cryo-MWCNT/n-Si photodetector consistently exhibits higher photocurrent values than the untreated C-MWCNT/n-Si device throughout the entire investigated wavelength range. The enhancement in photocurrent after cryogenic treatment suggests that the treatment effectively improves the electrical and interfacial properties of the MWCNT layer. Cryogenic processing may reduce structural defects, improve nanotube alignment, and enhance carrier mobility within the conductive network, thereby facilitating more efficient photogenerated charge carrier transport and collection. In addition, the improved interfacial contact between the MWCNT layer and the silicon substrate likely suppresses carrier recombination losses and promotes more efficient charge separation at the heterojunction interface.

The enhanced photocurrent response observed for the Cryo-MWCNT/n-Si device across the UV, visible, and NIR regions demonstrates that cryogenic treatment has a positive influence on the overall optoelectronic performance of the photodetector. The combination of broadband spectral sensitivity, self-powered operation, and enhanced photocurrent generation highlights the strong potential of the Cryo-MWCNT/n-Si heterojunction structure for advanced broadband photodetection applications. These findings indicate that cryogenic modification of the MWCNT interlayer is an effective strategy for improving the photoresponse characteristics and overall efficiency of carbon nanotube/silicon-based photodetectors.

**Fig. 11:**
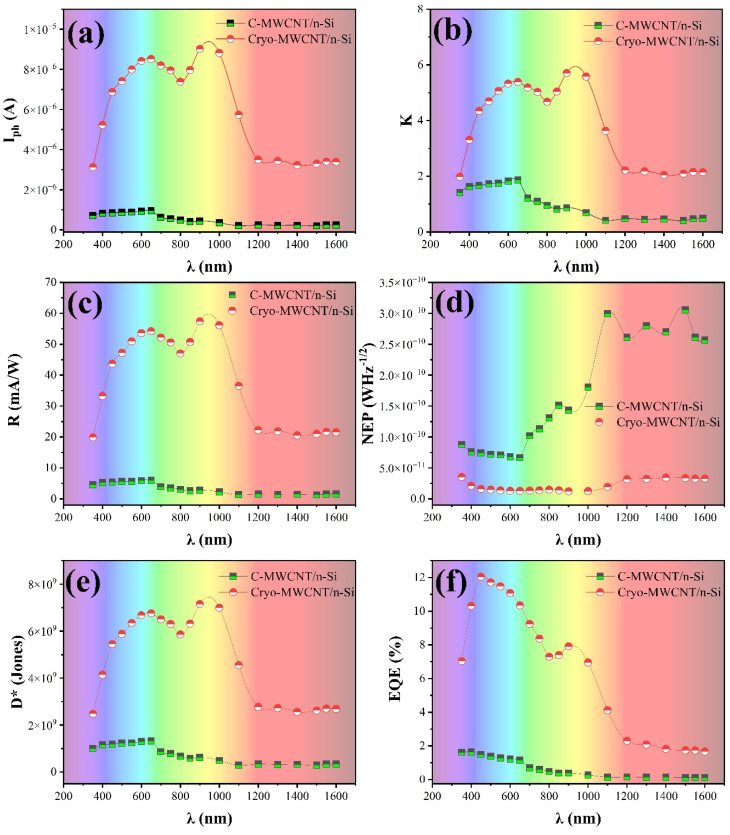
(a) *I*_ph_, (b) *K*, (c) *R*, (d) NEP, (e) D*, and (f) EQE plots of the devices under UV-Vis-NIR wavelengths.

Table 3 presents the spectral performance parameters of the C-MWCNT/n-Si and Cryo-MWCNT/n-Si photodetectors across a wavelength range of 351–1600 nm under self-powered conditions. These values were derived from the measured photocurrent responses obtained using narrow band-pass optical filters, which were then used to determine the main photodetection parameters. The calculated performance metrics include *I*_ph_, *K*, *R*, NEP, D*, and external quantum efficiency (EQE). The dependence of these parameters on wavelength is displayed in Fig. 11a–f, where the characteristics of photocurrent, photosensitivity, responsivity, NEP, D*, and EQE are illustrated, respectively.

**Table 3:** Photodetector parameters obtained at 351–1600 nm wavelengths

*λ* (nm)	C-MWCNT/n-Si				Cryo-MWCNT/n-Si			
	*R* (mA W^−1^)	D*	NEP	EQE	*R* (mA W^−1^)	D* (Jones)	NEP (WHz^−1/2^)	EQE (%)
		(Jones)	(WHz^−1/2^)	(%)				
351	4.59	1.01 × 10^9^	8.80 × 10^−11^	1.62	19.93	2.48 × 10^9^	3.57 × 10^−11^	7.04
400	5.29	1.16 × 10^9^	7.63 × 10^−11^	1.64	33.30	4.15 × 10^9^	2.14 × 10^−11^	10.32
450	5.42	1.19 × 10^9^	7.45 × 10^−11^	1.49	43.74	5.45 × 10^9^	1.63 × 10^−11^	12.05
500	5.61	1.23 × 10^9^	7.20 × 10^−11^	1.39	47.24	5.89 × 10^9^	1.51 × 10^−11^	11.71
550	5.67	1.24 × 10^9^	7.12 × 10^−11^	1.28	50.93	6.35 × 10^9^	1.40 × 10^−11^	11.48
600	5.92	1.30 × 10^9^	6.81 × 10^−11^	1.22	53.60	6.68 × 10^9^	1.33 × 10^−11^	11.08
650	6.05	1.33 × 10^9^	6.67 × 10^−11^	1.15	54.24	6.76 × 10^9^	1.31 × 10^−11^	10.35
700	3.95	8.67 × 10^8^	1.02 × 10^−10^	0.70	52.20	6.51 × 10^9^	1.36 × 10^−11^	9.25
750	3.57	7.84 × 10^8^	1.13 × 10^−10^	0.59	50.61	6.31 × 10^9^	1.40 × 10^−11^	8.37
800	3.09	6.77 × 10^8^	1.31 × 10^−10^	0.48	47.05	5.86 × 10^9^	1.51 × 10^−11^	7.29
850	2.67	5.85 × 10^8^	1.51 × 10^−10^	0.39	50.74	6.32 × 10^9^	1.40 × 10^−11^	7.40
900	2.81	6.17 × 10^8^	1.44 × 10^−10^	0.39	57.42	7.16 × 10^9^	1.24 × 10^−11^	7.91
1000	2.24	4.91 × 10^8^	1.80 × 10^−10^	0.28	56.15	7.00 × 10^9^	1.27 × 10^−11^	6.96
1100	1.35	2.96 × 10^8^	3.00 × 10^−10^	0.15	36.54	4.55 × 10^9^	1.95 × 10^−11^	4.12
1200	1.55	3.39 × 10^8^	2.61 × 10^−10^	0.16	22.28	2.78 × 10^9^	3.19 × 10^−11^	2.30
1300	1.44	3.17 × 10^8^	2.80 × 10^−10^	0.14	21.90	2.73 × 10^9^	3.25 × 10^−11^	2.09
1400	1.49	3.28 × 10^8^	2.70 × 10^−10^	0.13	20.56	2.56 × 10^9^	3.46 × 10^−11^	1.82
1500	1.32	2.90 × 10^8^	3.05 × 10^−10^	0.11	21.07	2.63 × 10^9^	3.37 × 10^−11^	1.74
1550	1.55	3.39 × 10^8^	2.61 × 10^−10^	0.12	21.71	2.71 × 10^9^	3.28 × 10^−11^	1.74
1600	1.57	3.45 × 10^8^	2.57 × 10^−10^	0.12	21.58	2.69 × 10^9^	3.29 × 10^−11^	1.67

The photocurrent of both devices shows a strong dependence on wavelength across the UV-visible (351–700 nm) and NIR (750–1600 nm) regions. In the UV-visible range, the C-MWCNT/n-Si device exhibits a gradual increase in photocurrent from 7.21 × 10^−7^ A at 351 nm to a maximum of 9.51 × 10^−7^ A at 650 nm, followed by a decrease to 6.21 × 10^−7^ A at 700 nm. In comparison, the Cryo-MWCNT/n-Si device shows significantly higher photocurrent values, increasing from 3.13 × 10^−6^ A at 351 nm to 8.52 × 10^−6^ A at 650 nm, and maintaining 8.20 × 10^−6^ A at 700 nm. In the NIR region, the photocurrent of the C-MWCNT/n-Si device decreases from 5.61 × 10^−7^ A at 750 nm to approximately 2.47 × 10^−7^ A at 1600 nm, while at 900 nm it reaches 4.42 × 10^−7^ A. Conversely, the Cryo-MWCNT/n-Si device maintains much higher photocurrent values throughout the NIR region, ranging from 7.95 × 10^−6^ A at 750 nm to 3.39 × 10^−6^ A at 1600 nm, with a value of 9.02 × 10^−6^ A at 900 nm, demonstrating enhanced photocarrier generation and collection efficiency compared with the untreated device.

The photosensitivity values also reveal a clear improvement in the performance of the Cryo-MWCNT/n-Si device. In the UV-visible region, the C-MWCNT/n-Si photodetector shows photosensitivity values increasing from 1.41 at 351 nm to 1.87 at 650 nm, then decreasing to 1.22 at 700 nm. In contrast, the Cryo-MWCNT/n-Si device exhibits much higher values, rising from 1.98 at 351 nm to 5.39 at 650 nm, and remaining high at 5.19 at 700 nm. In the NIR region, the photosensitivity of the C-MWCNT/n-Si device decreases significantly from 1.10 at 750 nm to approximately 0.48 at 1600 nm, while it is 0.867 at 900 nm. However, the Cryo-MWCNT/n-Si device maintains substantially higher photosensitivity, varying from 5.03 at 750 nm to about 2.15 at 1600 nm, with a value of 5.71 at 900 nm, indicating a much stronger light-response capability.

The responsivity values of the C-MWCNT/n-Si photodetector in the UV-visible region (351–700 nm) increase slightly from 4.59 mA W^−1^ at 351 nm to a maximum of 6.05 mA W^−1^ at 650 nm, followed by a decrease to 3.95 mA W^−1^ at 700 nm. In contrast, the Cryo-MWCNT/n-Si device exhibits significantly higher responsivity across the same region, rising from 19.93 mA W^−1^ at 351 nm to 54.24 mA W^−1^ at 650 nm, and remaining high at 52.20 mA W^−1^ at 700 nm. In the NIR region (750–1600 nm), the responsivity of the C-MWCNT/n-Si device decreases from 3.57 mA W^−1^ at 750 nm to approximately 1.57 mA W^−1^ at 1600 nm, while at 900 nm it is 2.81 mA W^−1^. Meanwhile, the Cryo-MWCNT/n-Si photodetector maintains much higher responsivity, decreasing gradually from 50.61 mA W^−1^ at 750 nm to 21.58 mA W^−1^ at 1600 nm, with a significantly higher value of 57.42 mA W^−1^ at 900 nm, indicating superior photoresponse performance.

The specific detectivity of the C-MWCNT/n-Si device in the UV-visible region ranges from 1.01 × 10^9^ Jones at 351 nm to a maximum of 1.33 × 10^9^ Jones at 650 nm, before decreasing to 8.67 × 10^8^ Jones at 700 nm. In comparison, the Cryo-MWCNT/n-Si device shows markedly higher detectivity values, increasing from 2.48 × 10^9^ Jones at 351 nm to 6.76 × 10^9^ Jones at 650 nm. In the NIR region, the detectivity of the C-MWCNT/n-Si device decreases significantly to about 2.90 × 10^8^–3.45 × 10^8^ Jones, while at 900 nm it is 6.17 × 10^8^ Jones. Conversely, the Cryo-MWCNT/n-Si device maintains detectivity in the order of 10^9^ Jones, decreasing from 6.31 × 10^9^ Jones at 750 nm to 2.69 × 10^9^ Jones at 1600 nm, with a value of 7.16 × 10^9^ Jones at 900 nm, confirming its superior detection capability.

The NEP values of the C-MWCNT/n-Si photodetector in the UV-visible region ranges from 8.80 × 10^−11^ to 6.67 × 10^−11^ WHz^−1/2^, indicating moderate sensitivity to weak optical signals. In contrast, the Cryo-MWCNT/n-Si device exhibits significantly lower NEP values, varying from 3.57 × 10^−11^ to 1.31 × 10^−11^ WHz^−1/2^, which reflects improved signal detection capability. In the NIR region, the NEP of the C-MWCNT/n-Si device increases to values as high as 3.05 × 10^−10^ WHz^−1/2^, while at 900 nm it is 1.44 × 10^−10^ WHz^−1/2^. Meanwhile, the Cryo-MWCNT/n-Si photodetector maintains relatively low NEP values, ranging from 1.40 × 10^−11^ to 3.29 × 10^−11^ WHz^−1/2^, with a value of 1.24 × 10^−11^ WHz^−1/2^ at 900 nm, confirming its enhanced sensitivity.

The external quantum efficiency of the C-MWCNT/n-Si photodetector decreases gradually across the spectral range. In the UV-visible region, the EQE decreases from 1.62% at 351 nm to 0.70% at 700 nm. In the NIR region, it further declines from 0.59% at 750 nm to approximately 0.12% at 1600 nm, while at 900 nm the EQE is 0.39%. In contrast, the Cryo-MWCNT/n-Si device shows significantly higher EQE values, ranging from 7.04% at 351 nm to a peak of 12.05% at 450 nm in the visible region, and gradually decreasing to 1.67% at 1600 nm in the NIR region, with a considerably higher value of 7.91% at 900 nm. These results demonstrate that the Cryo-MWCNT/n-Si photodetector achieves more efficient photocarrier generation and conversion across the entire spectral range.

The comparative analysis presented in Table 4 demonstrates that the Cryo-MWCNT/n-Si photodetector exhibits competitive optoelectronic performance among recently reported Si-based heterojunction photodetectors, particularly those incorporating MWCNT interlayers. The performance of MWCNT/Si photodetectors strongly depends on the incorporation of additional functional materials and the applied bias voltage.

**Table 4:** Comparison of MWCNT-based photodetector devices

Device	*λ* (nm)	*R* (A W^−1^)	D* (Jones)	EQE (%)	Applied bias (V)	References
MWCNT/Si	532	1.3 × 10^−3^	3.97 × 10^5^	0.29	—	57
	635	6.05 × 10^−3^	1.8 × 10^6^	1.14		
ZnS/MWCNTs/p-Si	610	0.67	3.37 × 10^11^	—	6.5	58
Ag NPs/MWCNTs/Si	532	17.7 × 10^−3^	—	—	5	59
Bi_2_O_3_-decorated MWCNTs/n-Si	560	1.37	—	3 × 10^2^	—	60
Ag/MWCNT/Ag	1550	0.284	—	22.7	0.5	61
	1064	0.264	—	30.8		
BATDS-11/n-Si	590	15.24	1.64 × 10^14^	4244	−2.0	62
Au/rGO/n-Si	590	0.43	1.06 × 10^12^	90.16	0	63
Si_3_N_4_/n-Si	351	1.59 × 10^−3^	2.49 × 10^8^	0.588	0	64
	500	4.65 × 10^−3^	7.28 × 10^8^	1.206		
	1000	8.73 × 10^−3^	1.36 × 10^9^	1.014		
Co_2_TiO_4_–ZnO/n-Si	351	17.39 × 10^−3^	8.61 × 10^9^	6.43	0	65
	550	21.34 × 10^−3^	1.06 × 10^10^	5.04		
	1000	23.76 × 10^−3^	1.18 × 10^10^	3.08		
Cryo-MWCNT/n-Si	450	43.74 × 10^−3^	5.45 × 10^9^	12.05	0	This work
	650	54.24 × 10^−3^	6.76 × 10^9^	10.35		
	900	57.42 × 10^−3^	7.16 × 10^9^	7.91		

A pristine MWCNT/Si device exhibited relatively low responsivities of 1.3 × 10^−3^ A W^−1^ at 532 nm and 6.05 × 10^−3^ A W^−1^ at 635 nm, with corresponding detectivities of 3.97 × 10^5^ and 1.8 × 10^6^ Jones, respectively.^57^ These values were obtained under an applied bias, indicating non-self-powered operation. The introduction of hybrid architectures generally leads to substantial performance enhancement but often requires relatively high operating voltages. For example, the ZnS/MWCNTs/p-Si photodetector achieved a responsivity of 0.67 A W^−1^ and a detectivity of 3.37 × 10^11^ Jones at 610 nm under a bias of 6.5 V.^58^ Similarly, the Ag nanoparticles/MWCNT/Si device exhibited a responsivity of 17.7 × 10^−3^ A W^−1^ at 532 nm under a 5 V bias, where plasmonic effects enhanced light absorption.^59^ The Bi_2_O_3_-decorated MWCNTs/n-Si photodetector further improved the responsivity to 1.37 A W^−1^ at 560 nm with an external quantum efficiency (EQE) of approximately 3 × 10^2^%, although the operating bias was not specified.^60^ In the near-infrared region, Ag/MWCNT/Ag photodetectors demonstrated responsivities of 0.284 A W^−1^ at 1550 nm and 0.264 A W^−1^ at 1064 nm, with EQE values of 22.7 and 30.8%, respectively, under a bias of 0.5 V.^61^

Compared with these MWCNT-based systems, the Cryo-MWCNT/n-Si photodetector developed in this work operates in a self-powered mode (0 V bias) while delivering responsivities of 43.74 × 10^−3^, 54.24 × 10^−3^, and 57.42 × 10^−3^ A W^−1^ at 450, 650, and 900 nm, respectively. Corresponding detectivities reach 5.45 × 10^9^, 6.76 × 10^9^, and 7.16 × 10^9^ Jones, with EQE values ranging from 7.91 to 12.05%. These results represent an improvement of several orders of magnitude over pristine MWCNT/Si devices and are comparable to or higher than those of other self-powered Si-based photodetectors employing dielectric interlayers, such as Si_3_N_4_/n-Si.

Although certain recently reported heterojunctions, including BATDS-11/n-Si and Co_2_TiO_4_–ZnO/n-Si, exhibit higher detectivities, these systems rely on more complex material architectures and, in the case of BATDS-11/n-Si, require an external bias of −2.0 V. In contrast, the Cryo-MWCNT/n-Si device combines a simple fabrication approach with broadband visible-to-near-infrared photoresponse and stable self-powered operation. Moreover, its detectivity exceeds that of the Au/rGO/n-Si photodetector operating at zero bias and approaches the performance range of more sophisticated oxide-based heterostructures. These findings highlight the effectiveness of cryogenic treatment in enhancing the interfacial properties of MWCNT/n-Si heterojunctions and demonstrate the potential of the proposed device for low-power, broadband photodetection applications.

## 4. Conclusion

In summary, C-MWCNT/n-Si and Cryo-MWCNT/n-Si Schottky photodetectors were successfully fabricated and their electrical and photoresponse characteristics were systematically investigated. The MWCNTs were first subjected to cryogenic treatment to produce Cryo-MWCNT, and the structural and surface properties of the modified nanotubes were examined using BET, XRD, Raman spectroscopy, SEM, and TEM analyses, confirming the successful modification of the nanotube structure. Electrical characterization revealed that the Cryo-MWCNT/n-Si device possesses improved diode parameters, including reduced series resistance and enhanced carrier transport, as determined from the thermionic emission model and Cheung's method. Under different illumination intensities, the Cryo-MWCNT/n-Si photodetector showed significantly higher photocurrent, photosensitivity, responsivity, and specific detectivity, together with lower NEP value, compared with the C-MWCNT/n-Si device. Time-dependent photoresponse measurements demonstrated stable and repeatable switching behavior with similar rise and fall times for both devices. In addition, broadband spectral analysis in the 351–1600 nm range under self-powered operation confirmed that the Cryo-MWCNT/n-Si device exhibits markedly enhanced performance across both the UV-visible and NIR regions. Overall, the improved photodetection performance is attributed to the enhanced electrical conductivity and improved interfacial properties introduced by the cryogenic treatment of MWCNTs. These findings suggest that Cryo-MWCNT interlayers provide an effective strategy for enhancing the performance of Si-based broadband photodetectors, offering strong potential for high-sensitivity and self-powered optoelectronic applications.

## Author contributions

Ali Akbar Hussaini: writing – review, characterization Dilber Esra Yıldız: writing – review and editing, characterization. Filiz Boran: writing – review and methodology. Murat Yıldırım: writing – review and editing, and characterization.

## Conflicts of interest

There is no conflict of interest between the authors.

## Data Availability

The data that support the findings of this study are available upon request from the authors.
